# Eye, Ear, Nose and Throat

**Published:** 1893-10

**Authors:** 


### EYE, EAR, NOSE AND THROAT.

EDITED BY DR. ARTHUR G. HOBBS, ASSISTED BY
DR. GEORGE BROWN.

### INJUDICIOUS NASAL OPERATIONS.

In opening the discussion on this subject at the late Pan-American Congress Dr. Hobbs said:

If our friends, the gynecologists, have their nymphomaniacs, I think we too may have our *rhinomaniacs*. We have a class (and it is a rapidly increasing class) of patients who consult us with the avowed purpose, to use their own language, "of having their noses burned out."

In some respects these two classes are analogous, in that the neurotic factor is often present also in our *rhinomaniacs*, as it is always present in nymphomaniacs.

My point is that we may, without much exaggeration, lay the blame of the injudicious, or at least the unnecessary operation, when we make it under such circumstances, upon the patient himself, unless we place it upon a higher plane, and refuse in all such cases even the pretense of an operation, as a placebo, or for its moral effect, when its necessity is not indicated. While judicious and properly executed rhinal operations have done, and are doing, an immense amount of good, for this very reason the temptation is increased in many instances to do operations that were better not to have been done, particularly by the younger men just making their entrée into this special line of work. They are loth to allow an opportunity to pass to saw a septum, to drill an exostosis, or to cauterize a turbinate. The mere suggestion of a septum spur, or a bony protuberance, or an enlarged turbinate is too often deemed a sufficient reason for a surgical procedure even, though no subjective or other objective symptoms may exist. When we are reminded of the large area of nasal mucous membrane, its functions should at once be suggested. The nasal space was intended to breathe through and only secondarily to serve as an olfactory organ.

As this great expansion of mucous surface, twice that of the so-called throat, is intended to perform the important functions of moistening and warming the inspired air, the necessity of its preservation should never be lost sight of. Hence any nasal operation made without reference to the mucous membrane preservation is not only injudicious but harmful, provided the nature of the case does not necessitate its destruction. And again, where no operation can be made without resulting in a large cicatrix the question may arise, "would it not be better left undone?"

In cases where chromic acid or any other escharotic would seem to be more appropriate on account of the timidity of the patient, or for any other reason, the toxic sequence should always be considered, otherwise the operation may prove to have been an injudicious one.

Septum deflections, spurs and exostoses should always be carefully measured before any operative procedure is resorted to, else a perforation may result that would be worse than the primary condition.

The drill is, in my opinion, almost always an injudicious device, for the reason that it necessarily destroys so much mucous membrane and leaves a rough cicatricial surface, although I find myself resorting to it in some cases of prominent exostoses.

To attempt to generalize upon the many injudicious external operations that are resorted to most frequently in malignant growths and occasionally in large benign tumors would require too much of your time, since each case of so serious a nature is a law unto itself and should always be so treated.

---

### EARACHE.

Dr. Alex. Randall, of Philadelphia (*American Journal of Med. Science*), sums up the treatment of earache as follows:

In conclusion, then, it may be repeated that earache is often due to acute tympanic inflammation arising from a naso-pharyngeal condition which demands treatment. Cleansing and detergent sprays and post-pharyngeal painting with astringents can control this and relieve any referred pain from this location. The hot syringing will give any needed cleansing, allay the local pain, and, by reducing the inflammatory congestion, help on the resolution. Protection, local and general, with medicinal treatment of general symptoms, will generally give such prompt and real relief that the host of other remedies may remain as an unemployed reserve. The physician summoned to a case of earache can generally leave his morphine and cocaine at home, if he will take his brow-mirror, a syringe and an atomizer.—*Memphis Med. Monthly.*

---

### TREATMENT OF NASAL STENOSIS BY INTRA-NASAL TUBES.

Gibbons (*N. Y. Medical Journal,* Vol. lvi., No. 2). Normal nasal respiration demands following conditions:

1. Free nasal passages.

2. Contact during inspiration and expiration between air and nasal mucous membrane.

3. A normal state of the nasal mucous membrane.

To satisfy these conditions in collapsed nares, all abnormal growths and deformities, so far as possible, should first be removed.

To maintain the patency of the nostril the author recommends metal tubes either flat or crescentic on cross section, made in fifteen sizes. The walls of the tubes are filled by minute perforations, allowing air and mucous membrane to come in contact. The metal may be gold, aluminum or silver; these are less irritating than rubber.

These tubes have proved very useful in stenosis of hay fever; before inserting them a thirty per cent. solution of cocaine should be used in the nose.

A sieve of metal or silk thread may be placed at end of tube and should be frequently moistened with antiseptic solution; this will moisten air inhaled, also prevent the entrance of dust or germs into the nasal cavity.—*Brooklyn Medical Journal.*

[Intra-nasal intubation is always objectionable and only in very rare cases at all practicable. However, as the author states, when nothing else remains to cause the stenosis except collapsed nares, his means of restoring nasal patency is as good if not better than any other.—ED.]

